# The Relationship between Constitution of Traditional Chinese Medicine in the First Trimester and Pregnancy Symptoms: A Longitudinal Observational Study

**DOI:** 10.1155/2016/3901485

**Published:** 2016-03-20

**Authors:** QiaoYu Jiang, Jue Li, GuangHua Wang, Jing Wang

**Affiliations:** ^1^Department of Prevention, Tongji University School of Medicine, Shanghai 200092, China; ^2^Department of Health Care Management, Public Health School, Fujian Medical University, Fuzhou, Fujian 350108, China; ^3^Key Laboratory of Arrhythmias, Ministry of Education, Tongji University School of Medicine, Shanghai 200092, China; ^4^Shanghai First Maternity and Infant Hospital, Tongji University School of Medicine, Shanghai 201204, China; ^5^Tongji Hospital, Tongji University, Shanghai 200065, China

## Abstract

*Objective*. We report on the distribution of traditional Chinese medicine (TCM) constitution in the first trimester and on the association between TCM constitution and maternal symptoms related to pregnancy.* Methods*. Participants were followed up until delivery to observe primary measures (gestational hypertension and gestational diabetes mellitus) and secondary measures (signs of miscarriage, miscarriage, nausea and vomiting, and sleepiness and defecation during pregnancy). Descriptive analysis,* t*-tests, chi-square tests, and logistic regression analysis were used in this study.* Results*. 61.8% of the participants had unbalanced constitutions. We did not find a significant association between the TCM constitution and gestational hypertension, gestational diabetes, miscarriage, signs of miscarriage, and defecation during pregnancy. And we found that women with unbalanced constitutions in early pregnancy had a greater likelihood of severe nausea and vomiting and poor sleep during pregnancy in the logistic regression analysis.* Conclusions*. These results have implications for female health care providers and policy makers. Identification of TCM constitution may be helpful for understanding nausea and vomiting and poor sleepiness during pregnancy, especially in the condition that can not be explained by modern medical science, and be helpful for making program to improve these uncomfortable symptoms.

## 1. Introduction 

With pregnancy and the birth of every infant, women can suffer from many physiological changes. These changes may predispose them to develop uncomfortable symptoms and adverse outcomes, such as fatigue, dizziness, sleep disorders, nausea and vomiting, and defecation disorders. Many studies have tried to determine which risk factors lead to these complications [1, 2]. In most countries, perinatal care [3] has been applied to reduce the incidence of pregnancy related adverse events. In perinatal care, there are several unanswered questions: why do women with similar demographic and medical characteristics, as assessed by modern medicine, have different pregnancy symptoms and outcomes? That is, why do some women maintain comfortable and favorable conditions during pregnancy while others develop uncomfortable and adverse events? How can we provide personalized health care services for women with similar demographic and medical characteristics?

The Chinese concept of constitution (i.e., the constitution of traditional Chinese medicine (TCM)) is an indigenous construct that serves as part of an explanatory model for understanding various aspects of life, including physical well-being. Constitution (i.e.,* ti-zhi*) is a widely used term in China. Literally,* ti* means body and* zhi* denotes quality or substance. TCM takes a global and dynamic view of human differences, believes that constitution is partly genetically determined and partly acquired, and classifies individuals' constitution into nine types based on Chinese medical theory, multidisciplinary studies, and clinical practice. According to the shape of the human body, function, psychology, and other characteristics, individual constitution can be assessed by the Constitution in Chinese Medicine Questionnaire (CCMQ) developed by Wang et al. [4–6]. One type is balanced constitution (i.e., a normal constitution), and the following eight types represent unbalanced constitution: Qi-deficiency constitution, Yang-deficiency constitution, Yin-deficiency constitution, Phlegm-dampness constitution, Damp-heat constitution, Stagnant Blood constitution, Stagnant Qi constitution, and Inherited Special constitution. Unbalanced constitution means disharmony and can be viewed as an individual's susceptibility to specific disease or symptoms.

According to TCM theory, women with unbalanced constitutions are at higher risk of uncomfortable symptoms and adverse outcomes because they cannot adapt well to the physiological and psychological changes accompanying pregnancy and delivery. Qi and Blood deficiency are disadvantages of menstruation, pregnancy, and childbearing and influence both maternal health and fetal/newborn health [7, 8].

Prenatal care and neonatal care have been used to prevent and reduce adverse pregnant outcomes [9–11]. Preconception care also was used to supplement and increase the likelihood of a desire and healthy pregnancy and a healthy infant by providing timely and exact information and intervention [12, 13]. However, women are satisfied with common preconception and prenatal care based on the modern medical concept of health status; therefore, personalized health care services would be more acceptable. The theory of TCM constitution provides personalized services for all women in the following three aspects. First, people have different constitutions even if they have similar demographic and medical characteristics [14]; second, different constitutions require different treatments (i.e., different food due to different food properties, different guidance on work and rest and on exercise regimen, and different Chinese herbs) [5, 15, 16]; third, different foods that provide similar nutrients may not have the same TCM natures, so due to different food properties, people should choose better foods according to their constitutions [5, 15, 16]. For example, people with Yang-deficiency are recommended to eat food that can warm the Yang, such as ginger, chestnut, walnut, chives, beef, and mutton, and to avoid uncooked and cold food, such as pear, watermelon, grapefruit, raw vegetable salad, cold milk, cold beer and ice cream, and green tea. They should keep warm, especially the feet, upper back, and lower abdomen, particularly in autumn and winter, and ventilate the living room. Proper outdoor activities on sunny days are recommended for Yang-deficiency, whereas strenuous exercise in the summer and under adverse environmental conditions (i.e., strong wind, bitter cold, heavy fog, heavy snow, or air pollution) is not suggested.

In recent years, accumulating data have demonstrated that TCM plays an important role in health care [17] and disease prevention and treatment [14, 18, 19]. Complementary treatments were always more acceptable among women [17, 20] and were used for preconception care [21] not only in China. From the TCM perspective, food not only cures hunger but also has medicinal properties. Food, as well as Chinese herbs and acupuncture, is helpful for improving health if the right foods are selected according to individual TCM constitutions [5, 15]. Therefore, it is feasible to integrate the TCM constitution theory into female health care. The application of TCM/TCM constitution would bring about more personalized services during perinatal care. And, how to apply TCM/TCM constitution theories into perinatal care requires more basic data, such as the association between TCM constitution and maternal discomfort and pregnancy outcomes.

There have been few published reports on the relationship between TCM constitution and maternal symptoms during pregnancy. The aim of this study was to assess potential associations between the TCM constitution in the first trimester and maternal symptoms during pregnancy.

## 2. Materials and Methods

This prospective observational study was conducted at the Zhabei District Maternity and Child Care Center in Shanghai, China. The Ethics Committee of Tongji University Medicine and Life Science Unit specifically approved this study (Number 2013-yxy07), and written informed consent was obtained from all participants prior to study initiation.

**Figure 1:**
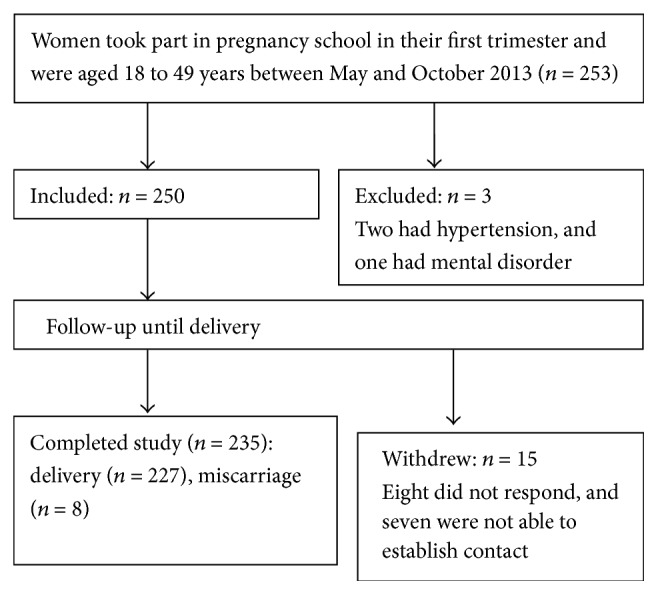
Study profile.

Women who participated in Pregnancy School held by the Zhabei District Maternity and Child Care Center between May and October 2013, who are aged 18 to 49 years, and whose gestational time was less than or equal to 12 weeks were asked to take part in an investigation of baseline information and evaluation of their TCM constitution using the Constitution in Chinese Medicine Questionnaire (CCMQ) [4, 6]. And then women included in this study were followed up until delivery to observe the maternal symptoms (Figure 1).

Women who refused to answer the questionnaire, who had hypertension, who had diabetes, who had somnipathy, and who had mental disorder were excluded from the study.

### 2.1. Study Protocol

To explore the relationship between the TCM constitution in the first trimester and maternal symptoms during pregnancy, a longitudinal study protocol was undertaken. The TCM constitution was determined face to face at the first trimester pregnancy. Women were followed up until delivery.

### 2.2. Data Collection

The baseline information and TCM constitution were examined when women took part in the free pregnancy school held for pregnant women in their first trimesters. Gestational hypertension (GH) and gestational diabetes mellitus (GDM) were collected from women's medical record. Nausea and vomiting, signs of a miscarriage, miscarriage, sleepiness, and defecation were recorded at the follow-up phone call. Two study investigators performed data collection after training.

### 2.3. Measures

TCM constitution: the Constitution in Chinese Medicine Questionnaire (CCMQ) [4, 6] was used to examine women's TCM constitutions. CCMQ, developed by Wang et al. [4–6], is a 60-item, 5-point Likert scale (from 1 (almost not) to 5 (always happen)). It is composed of nine independent constitution subscales including one balanced constitution and eight unbalanced constitutions: Qi-deficiency, Yang-deficiency, Yin-deficiency, Phlegm-dampness, Damp-heat, Stagnant Blood, Stagnant Qi, and Inherited Special constitutions. If the subscale of balanced constitution scores greater than or equal to 60 and all the remaining eight subscales score less than 40, then balanced constitution was determined. If the subscale of Qi-deficiency constitution scores greater than or equal to 40, then Qi-deficiency was determined; and the same determination method was applied to Yang-deficiency, Yin-deficiency, Phlegm-dampness, Damp-heat, Stagnant Blood, Stagnant Qi, and Inherited Special constitutions. The reproducibility of CCMQ ranged from 0.76 to 0.90 for 9 subscales, Cronbach's *α* in each subscale was between 0.72 and 0.80, and the balanced constitution measured by CCMQ was positively corrected with SF-36 (*r* = 0.58, *p* < 0.01), while the unbalanced constitutions were negatively corrected with SF-36 (*r* = 0.38 ~ 0.54, *p* < 0.01) [4–6].

Primary observations included gestational hypertension (GH) and gestational diabetes mellitus (GDM).

GH in this study means that women did not have hypertension before pregnancy and had high blood pressure (SBP ≥ 140 mmHg and/or DBP ≥ 90 mmHg) that developed after pregnancy.

GDM women at 24 to 28 weeks of gestation have the diagnostic fasting 2-hour 75 g oral glucose tolerance test (75 g OGTT). The test is interpreted as positive for GDM if at least one of the values exceeds its corresponding thresholds (Table 1).

Secondary observations included nausea and vomiting, signs of a miscarriage, miscarriage, sleepiness during pregnancy, and defecation during pregnancy.

Nausea and vomiting were classified into four levels according to the length of influence on the normal diet: none (=1), within one week (=2), between one week and two weeks (=3), and more than two weeks (=4).

Signs of a miscarriage were identified by the following questions: (1) Did you have abdominal pain and/or vaginal bleeding during early pregnancy? (2) Did you go to see the doctor? (3) Did the doctor diagnose these symptoms as signs of miscarriage? If the response was “yes” to all three questions, “yes” was recorded; otherwise “no” was recorded.

Miscarriage was classified into two levels according to women's reply. If women reply that they lost the fetus before 28 weeks, “yes” was recorded; otherwise, “no” was recorded.

Sleepiness during pregnancy was observed in this study. According to the Pittsburg Sleep Quality Index (PSQI) [22], women were asked the following questions: (1) Can you fall asleep within 30 minutes? (2) Can you sleep without waking up in the middle of the night or early morning? (3) Can you get 6–8 hours of actual sleep at night? (4) Do you feel active, vital, alert, or wide awake during the day? If women responded “yes” to all four questions for the entire length of pregnancy, “good” was recorded (=1). If women responded “no” during more than 25% of the pregnancy to one or more of the above questions, “poor” was recorded (=3). Otherwise, “moderate” was recorded (=2).

Defecation during pregnancy was observed in this study. If women described 1-2 defecations per day and persistent loose stools without the use of laxatives, “good” was recorded (=1). According to the ROME III criteria [23, 24], if women described straining during more than 25% of defecations, lumpy or hard stools in more than 25% of defecations, and/or less than three defecations per week, “poor” was recorded (=3). Otherwise, “moderate” was recorded.

### 2.4. Analysis

The data were analyzed using SAS 9.2 Software. Statistical significance was defined as *p* < 0.05 (two-tailed test). Descriptive analyses were performed. In the final analysis, TCM constitution was classified into two groups (i.e., the balanced constitution group and the unbalanced constitution group). The *t*-test was used to compare the two groups for continuous variables, and the chi-square test was used for categorical variables. We executed logistic regression analysis to reveal the relationship between outcome observations which were significantly different in the two groups in the chi-square test/*t*-test and TCM constitution and controlled for the baseline variables (i.e., job stress, interpersonal relationship stress, economic stress, and aversions to vegetable) because of their significant difference between the balanced group and the unbalanced group. Two models were built in the logistic regression analysis, and the dependent variables, respectively, are nausea and vomiting (according to whether they influence diet, no = 1, yes = 2) and sleepiness during pregnancy (poor = 2, good or moderate = 1).

## 3. Results

### 3.1. Baseline Characteristics

**Figure 2:**
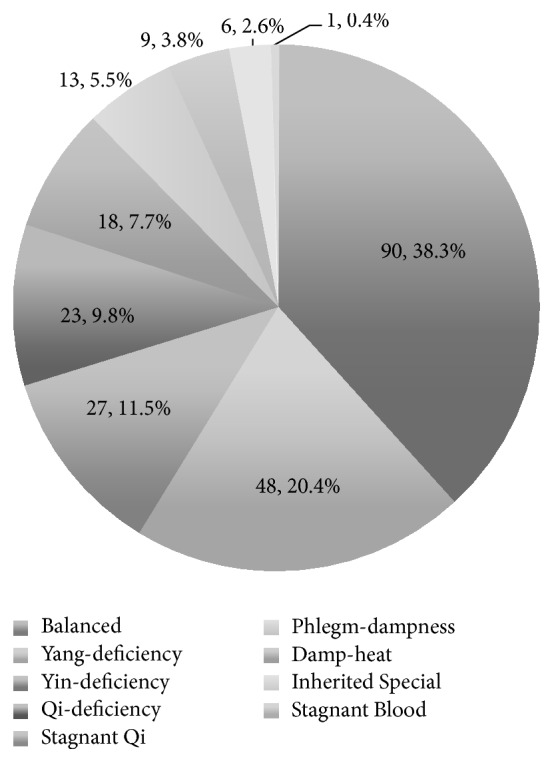
The distribution of the TCM constitutions in early pregnancy.

A total of 235 women completed the study. They were aged 22 to 38 years (mean ± SD, 28.35 ± 2.75). The balanced constitution group included 90 participants (38.3%), and the unbalanced constitution group included 145 participants (61.7%; i.e., Yang-deficiency (48, 20.4%), Yin-deficiency (27, 11.5%), Stagnant Qi (23, 9.8%), Qi-deficiency (18, 7.7%), Phlegm-dampness (13, 5.5%), Damp-heat (9, 3.8%), Inherited Special (6, 2.6%), and Stagnant Blood (1, 0.4%)) (Figure 2).

No woman included in this study had hypertension, diabetes, epilepsy, nephritis, malignancy, hepatitis B, and mental disorder.

Women's education was classified into four levels: middle school and below, high school, college, and graduate and above. The household income per capita was categorized into six levels: 1000 RMB and below, 1001 to 2000 RMB, 2001 to 3000 RMB, 3001 to 4000 RMB, 4001 to 5000 RMB, and 5001 RMB and above. There were no significant differences (*p* = 0.74, *p* = 0.99, resp.) in education and household incomes per capita between the groups. The remaining baseline characteristics are summarized in Table 1. Job stress, interpersonal relationship stress, economic stress, and aversions to vegetable were found significantly different between the two groups in baseline test (Table 2).

### 3.2. The Analysis of the Primary Observations

There were no significant differences in the GH and GDM (Table 3).

### 3.3. The Analysis of Secondary Observations during Pregnancy

Women with unbalanced constitution underwent more severe nausea and vomiting (*χ* ^2^ = 7.47, *p* = 0.01) (Table 4), especially among the women aged greater than or equal to 30 years. Women with unbalanced constitution experienced poorer sleep during pregnancy (*χ* ^2^ = 8.22, *p* = 0.00) (Table 4), especially among the women aged less than 30 years. There were no significant differences among the signs of miscarriage, miscarriage, and defecation during pregnancy (Table 4).

### 3.4. The Logistic Regression Analysis of Pregnant Outcomes

Because the baseline variables (i.e., job stress, interpersonal relationship stress, economic stress, and aversions to vegetable) were found significantly different between the two groups and the observations (i.e., nausea and vomiting and sleepiness during pregnancy) were found significantly and negatively associated with the balanced constitution, we built two models in the logistic regression analysis controlled for job stress, interpersonal relationship stress, economic stress, and aversions to vegetable. It was showed that, compared to balanced constitution, unbalanced constitution easily brings about nausea and vomiting influencing diet (OR, 95% CI; 2.58, 1.38–4.82) and poor sleep during pregnancy (OR, 95% CI; 2.24, 1.17–4.29) (Table 5).

## 4. Discussion

In this study, about sixty-two percent of women in the first trimester had unbalanced constitution. There was no significant association between the TCM constitution and the primary observations (GH and GDM). No significant associations between TCM constitution and signs of a miscarriage, miscarriage, and defecation during pregnancy were found. But we found that women with unbalanced constitutions in early pregnancy had a greater likelihood of severe nausea and vomiting and poor sleep during pregnancy.

High incidence rate of GDM was found in unbalanced TCM constitution group (i.e., 9.2% versus 3.5%), but no significant difference (*p* = 0.10) was found in this study.

Miscarriage is defined as the spontaneous loss of an intrauterine pregnancy that occurs before the fetus can survive outside the uterus. In modern medicine, cytogenetic abnormalities (50%–85%), antiphospholipid syndrome, inherited thrombophilia, and congenital structural abnormalities of the uterus were found to be the main causes of miscarriage [25, 26]. The risk of miscarriage is also increased in women with cardiovascular disease [27], disease of the thyroid gland [28], and obesity [29]. In this study, medication and the prevalence of thyroid disease, diabetes, and BMI were balanced among three constitution groups. Eight women (3.40%), with no detected genes or biological risks, suffered from miscarriage in this study. No significant differences were found between the two groups. This result may be due to the small sample size of this study. Vaginal bleeding and loss of pregnancy symptoms are suggestive of miscarriage. There were also no significant differences in the sign of the first miscarriage in this study.

Nausea and vomiting, beginning in the first trimester at about six to eight weeks' gestation and typically peaking at about nine weeks' gestation and returning to baseline by 12 weeks, are the most common symptoms of pregnancy and affect 50–90% of pregnant women [30]. However, severe and protracted nausea, vomiting, and discomfort may cause a poor intake of necessary nutrients and further affect maternal and fetal health. In the cross-sectional study, Kuo et al. [31] did not find significant association between nausea and Tan-Shi-Yu-Zhi (a kind of unbalanced constitution) in the first trimester. In this longitudinal observational study, we found that women with unbalanced constitutions in early pregnancy had more opportunities to have severe nausea and vomiting (influencing normal diet). Thus, women with unbalanced constitutions need more attention and information on how to avoid or lessen severe nausea and vomiting during perinatal care. If the classification and adjustment of TCM constitution can be conducted in prepregnancy and/or early pregnancy, severe nausea and vomiting might be avoided and lessened. It also implied that treatment based on the TCM constitution would be effective in alleviating nausea and vomiting.

All of the women did not have organic causes for poor defecation during pregnancy in this study. The constipation prevalence rates were 11%–24% during pregnancy and 24% at 3 months postpartum [32, 33]. The pathophysiology underlying functional constipation during pregnancy is undoubtedly multifactorial and not well understood. It was reported that progressively rising progesterone and estrogen levels have been suggested to be causes of constipation during pregnancy [34], and iron supplements and past constipation treatment were associated with constipation during pregnancy [33]. In this study, we did not find a significant association between the TCM constitution and defecation during pregnancy.

Sleep is a fundamental neurobehavioral state linked to critical domains of health and functioning, including learning and memory consolidation, mood, and disease risk [35]. And women with the worst subjective sleep quality during pregnancy were also the most likely to experience high symptoms of depression in the postpartum period [36]. There is a long history of investigation into the implications of sleep loss and sleepiness of pregnant/postpartum women [37–39]. It was explored that sleep of pregnancy/postpartum was associated with prepregnancy BMI and depressive and fatigue symptoms during third-trimester pregnancy [40]. It has been reported that infant feeding methods were not associated with objective, subjective, or sleepiness/fatigue [41]. Additionally, the baseline characteristics (i.e., job stress, interpersonal relationship stress, economic stress, and aversions to vegetable) have been adjusted in the logistic regression analysis. The finding that women with unbalanced constitutions had less proportion of good sleepiness during pregnancy implies that identification of TCM constitutions may be helpful for understanding or improving sleepiness during pregnancy.

This longitudinal protocol study is one of the first to explore the relationship between TCM constitution in the first trimester and maternal symptoms during pregnancy. There are some limitations in this study. First, recall bias in observations during pregnancy may potentially jeopardize the validity of the epidemiologic results. Second, in this study, to understand the condition of sleepiness and defecation throughout pregnancy, besides considering the Pittsburg Sleep Quality Index (PSQI) and ROME III criteria, we also considered the frequency of events for judging the level of these two observations. The definition of these two observations may reduce comparability with other reports. Third, this is not a large sample study. A small sample size may affect the reliability. Additionally, because of the small size sample, we can not perform multilevel stratified analysis by age and can not explore the further relationship between the specific type of unbalanced constitution and pregnancy symptoms. Hence, future research could include larger sample to improve reliability of results and obtain more evidence-based data. Fourth, we did not report on TCM constitution in the second and the third trimester, which may be associated with pregnancy symptoms. Hence, future research should observe the evolution of TCM constitution during pregnancy to provide more suitable evidence-based data.

## 5. Conclusion

About sixty-two percent of women had unbalanced constitutions in the first trimester. We did not find a significant association between the TCM constitution and miscarriage, signs of a miscarriage, and defecation during pregnancy in the logistic regression analysis. However, women with unbalanced constitutions in early pregnancy had a greater likelihood of severe nausea and vomiting and poor sleepiness during pregnancy. Identifying and adjusting the TCM constitutions of women during perinatal care, maybe better during preconception care, will help understand and improve nausea and vomiting and sleepiness during pregnancy.

## Supplementary Material

Appendix 1: multiple logistic regression analysis (vomiting was considered as independent factor).
Appendix 2: stratified analysis by age subgroups (less than 30 years and greater or equal to 30 years).

## Figures and Tables

**Table 1:** The 75 g OGTT thresholds for the diagnosis of gestational diabetes.

Definitive test	Fasting 2-hour 75 g oral glucose tolerance test (OGTT)
Fasting threshold	≥92 mg/dL (5.1 mmol/L)
1-hour threshold	≥180 mg/dL (10.0 mmol/L)
2-hour threshold	≥153 mg/dL (8.5 mmol/L)

**Table 2:** Baseline characteristics of the balanced and unbalanced constitution groups.

Variable	Constitution group		*χ* ^2^/*t*	*p*
	Balanced (*n* = 90)	Unbalanced (*n* = 145)		
Family registered in Shanghai (yes), *n* (%)	69 (76.7)	110 (75.49)	0.02	0.89
Prior pregnancy (yes), *n* (%)	22 (24.4)	35 (24.1)	0.00	0.96
Prior adverse pregnancy outcomes (yes), *n* (%)	5 (5.6)	16 (11.0)	2.05	0.15
Irregular menstrual cycle (yes), *n* (%)	12 (13.3)	21 (14.5)	0.06	0.81
Medication (yes), *n* (%)	16 (17.8)	21 (14.5)	0.45	0.50
Anemia (yes), *n* (%)	11 (12.2)	13 (9.0)	0.64	0.42
Thyroid disease (yes), *n* (%)	4 (4.4)	4 (2.8)		0.49^#^
Heart disease (yes), *n* (%)	0 (0.0)	1 (0.7)		1.00^#^
Gum bleeding (yes), *n* (%)	32 (36.0)	55 (38.2)	0.12	0.73
Aversions to meat/egg (yes), *n* (%)	1 (1.1)	7 (4.9)		0.16^#^
**Aversions to vegetable (yes)**, *n* (%)	**3 (3.4)**	**17 (11.8)**	**4.99**	**0.03**
Preferences for raw meat (yes), *n* (%)	4 (4.4)	6 (4.2)		1.00^#^
Smoke (yes), *n* (%)	2 (2.2)	3 (2.1)		1.00^#^
Drink (yes), *n* (%)	0 (0.0)	3 (2.1)		0.29^#^
Prenatal risk (yes), *n* (%)	17 (18.9)	37 (25.5)	1.38	0.24
Dysmenorrheal, *n* (%)			2.69	0.26
Much	11 (12.2)	22 (15.2)		
Moderate	43 (47.8)	80 (55.2)		
**Stress from job**, *n* (%)			**12.41**	**0.00**
Much	5 (5.6)	15 (10.3)		
Moderate	49 (54.4)	102 (70.3)		
**Economic stress**, *n* (%)			**7.64**	**0.02**
Much	1 (1.1)	8 (5.5)		
Moderate	35 (38.9)	74 (51.0)		
**Stress from interpersonal relationship^*∗*^** Moderate, *n* (%)	**16 (17.8)**	**49 (33.8)**	**7.12**	**0.01**
Defecation during prepregnancy^*∗∗*^, *n* (%)			0.20	0.65
Good	84 (93.3)	133 (91.7)		
Moderate	6 (6.7)	12 (8.3)		
Poor	0	0		
Sleepiness during prepregnancy^*∗∗*^, *n* (%)			0.92	0.34
Good	62 (68.9)	91 (62.8)		
Moderate	28 (31.1)	54 (37.2)		
Poor	0	0		
Age (year), mean ± SD	28.55 ± 2.68	28.23 ± 2.80	0.89	0.38
BMI (kg/m^2^), mean ± SD	21.28 ± 2.82	20.62 ± 2.34	1.88	0.06
SBP (mmHg), mean ± SD	108.0 ± 9.75	106.4 ± 11.15	1.11	0.27
DBP (mmHg), mean ± SD	68.75 ± 7.14	67.64 ± 6.89	0.18	0.24

Note: data are presented as *n* (%) and *χ* ^2^-value for categorical variables and mean ± SD and *t*-value for continuous variables.

^#^Fisher's exact test; ^*∗*^no women felt much stress from interpersonal relationship; ^*∗∗*^six months before pregnancy.

**Table 3:** Primary observations evaluated by the chi-square test.

Variable	Constitution group		*χ* ^2^	*p*
	Balanced (*n* = 90)	Unbalanced (*n* = 145)		
Gestational hypertension (yes), *n* (%)	2 (2.3)	1 (0.7)		0.56^#^
Gestational diabetes (yes), *n* (%)	3 (3.5)	13 (9.2)	2.68	0.10

^#^ *p* value of Fisher's exact test.

**Table 4:** The secondary observations assessed by the chi-square test.

Variable	Constitution group		*χ* ^2^/*t*	*p*
	Balanced (*n* = 90)	Unbalanced (*n* = 145)		
**Nausea and vomiting influencing diet**, *n* (**%**)			**7.47^**∗**^**	**0.01**
None	35 (38.9)	27 (18.6)		
<1 week	15 (16.7)	32 (22.1)		
1 week-2 weeks	29 (32.2)	64 (44.1)		
>2 weeks	11 (12. 2)	22 (15.2)		
Sign of miscarriage (yes), *n* (%)	18 (20.5)	27 (18.9)	0.09	0.77
Miscarriage (yes), *n* (%)	4 (4.4)	4 (2.8)		0.49^#^
**Sleepiness during pregnancy**, *n* (**%**)			**8.22^**∗**^**	**0.00**
Good	67 (77.9)	86 (61.0)		
Moderate	19 (22.1)	50 (35.5)		
Poor	0 (0.0)	5 (3.6)		
Defecation during pregnancy, *n* (%)			0.52^**∗**^	0.47
Good	73 (84.9)	115 (81.6)		
Moderate	12 (14.0)	23 (16.3)		
Poor	1 (1.2)	3 (2.1)		

^#^ *p* value of Fisher's exact test; ^*∗*^value of Mantel-Haenszel chi-square.

**Table 5:** The logistic models of nausea and vomiting and sleep during pregnancy.

Variables	*β*	SE	Wald	*p*	OR	95% CI
*Model 1*						
**TCM constitution**	**0.47**	**0.16**	**8.79**	**0.00**	**2.58**	**(1.38, 4.82)**
Job stress	0.36	0.32	1.24	0.27	1.43	(0.76, 2.70)
Interpersonal relationship stress	0.45	0.41	1.18	0.28	1.56	(0.59, 2.13)
Economic stress	0.11	0.33	0.12	0.73	2.58	(0.70, 3.49)
Aversions to vegetable	0.36	0.28	1.79	0.18	2.08	(0.71, 6.07)
*Model 2*						
**TCM constitution**	**0.40**	**0.17**	**5.94**	**0.01**	**2.24**	**(1.17, 4.29)**
Job stress	−0.08	0.31	0.07	0.79	0.92	(0.50, 1.70)
**Interpersonal relationship stress**	**1.25**	**0.36**	**12.10**	**0.00**	**3.48**	**(1.72, 7.03)**
Economic stress	−0.35	0.32	1.23	0.27	0.70	(0.38, 1.31)
Aversions to vegetable	0.55	0.31	3.19	0.07	3.01	(0.90, 10.08)

Model 1: nausea and vomiting influencing diet are the dependent variable.

Model 2: sleepiness during pregnancy is the dependent variable.
