# Supplementary material for: The Relationship between Constitution of Traditional Chinese Medicine in the First Trimester and Pregnancy Symptoms: A Longitudinal Observational Study
Source: Evid Based Complement Alternat Med. 2016 Mar 20;2016:3901485. doi: 10.1155/2016/3901485 (PMC4818819; doi:10.1155/2016/3901485)
Supplement: Supplementary file 1 — Appendix 1: multiple logistic regression analysis (vomiting was considered as independent factor). Appendix 2: stratified analysis by age subgroups (less than 30 years and greater or equal to 30 years). [file 3901485.f1.zip › Appendices.pdf]

Appendix 1 multiple logistic regression analysis (vomiting was considered as independent factor)

Table 1 the result of logistic regression analysis.

Variables	β	SE	Wald	*P*	OR	95%CI
**Vomiting influencing diet**	**0.88**	**0.41**	**4.50**	**0.03**	**2.40**	**(1.07, 5.38)**
Job stress	0.28	0.39	0.51	0.48	1.32	(0.62, 2.83)
Interpersonal relationship stress	0.64	0.49	1.73	0.19	1.89	(0.73, 4.90)
Economic stress	0.24	0.41	0.35	0.56	1.27	(0.57, 2.83)
Aversions to vegetable	1.92	1.09	3.08	0.08	6.80	(0.80, 57.76)

TCM constitution is the dependent variable (balanced constitution =1, unbalanced constitution=2). Adjusted variables included vomiting influencing diet, job stress, interpersonal relationship stress, economic stress, and aversions to vegetable.

Table 2 Results of Multinomial Regression Analysis Comparing Unbalanced Constitution Groups to Balanced Constitution Group

Factors	Qi-deficiency (n=23)		Yang-deficiency (n=48)		Yin-deficiency (n=27)		Phlegm-dampness (n=13)		Damp-heat (n=9)		Stagnant Blood (n=1)		Stagnant Qi (n=18)		Inherited special (n=6)	
	OR	95%CI	OR	95%CI	OR	95%CI	OR	95%CI	OR	95%CI	OR	95%CI	OR	95%CI	OR	95%CI
Vomiting influencing diet	2.60	0.86-7.90	**2.43***	**1.15-5.13**	1.46	0.61-3.48	2.25	0.65-7.84	3.50	0.69-17.78	<0.001		1.30	0.52-3.27	1.00	0.19-5.22

The reference group is Balanced Constitution (N=90). AOR, adjusted odds ratio; CI, confidence interval. *p<0.05.

Appendix 2 stratified analysis by age subgroups (less than 30 years and greater or equal to 30 years)

Table 1 Primary observations evaluated by the chi-square test (age less than 30 years, n=166).

Variable	Constitution group		χ^2	P
	Balanced (n=60)	Unbalanced (n=106)		
Gestational hypertension (yes), n (%)	1(1.7)	1(0.9)		1.00[#]
Gestational diabetes (yes), n (%)	2(3.4)	10(9.5)		0.21[#]

[#] P-value of Fishers exact test.

Table 2 Primary observations evaluated by the chi-square test (age greater or equal to 30 years, n=69).

Variable	Constitution group		χ^2	P
	Balanced (n=30)	Unbalanced (n=39)		
Gestational hypertension (yes), n (%)	2(7.4)	0(0.0)		0.56[#]
Gestational diabetes (yes), n (%)	1(3.7)	3(8.3)		0.63[#]

[#] P-value of Fishers exact test.

Table 3 The secondary observations assessed by the chi-square test (age less than 30 years, n=166).

Variable	Constitution group		χ^2/t	P
	Balanced (n=60)	Unbalanced (n=106)		
Nausea and vomiting influencing diet, n (%)			2.83*	0.09
None	20(33.3)	22(20.8)		
< 1 week	10(16.7)	24(22.6)		
1week-2weeks	24(40.0)	43(40.6)		
>2weeks	6(10.0)	17(16.0)		
Sign of miscarriage (yes), n (%)	12(20.0)	20(19.5)	0.02	0.88
Miscarriage (yes), n (%)	1(1.7)	1(0.9)		1.00#
**Sleepiness during pregnancy, n (%)**			**7.69***	**0.01**
Good	47(79.7)	63(60.0)		
Moderate	12(20.3)	37(35.2)		
Poor	0(0.0)	5(4.8)		
Defecation during pregnancy, n (%)			0.44*	0.51
Good	50(84.8)	85(81.0)		
Moderate	8(13.6)	17(16.0)		
Poor	1(1.7)	3(2.6)		

# P-value of Fishers exact test. * value of Mantel-Haenszel Chi-Square.

Table 4 The secondary observations assessed by the chi-square test (age greater or equal to 30 years, n=69).

Variable	Constitution group		χ^2/t	P
	Balanced (n=30)	Unbalanced (n=39)		
**Nausea and vomiting influencing diet, n (%)**			**5.50***	**0.02**
None	15(50.0)	5(12.8)		
< 1 week	5(16.7)	8(20.5)		
1week-2weeks	5(16.7)	21(53.8)		
>2weeks	5(16.7)	5(12.8)		
Sign of miscarriage (yes), n (%)	6(21.4)	7(18.4)	0.09	0.76
Miscarriage (yes), n (%)	3(2.6)	3(3.4)		1.00#
**Sleepiness during pregnancy, n (%)**			**0.74**	**0.39**
Good	20(74.1)	23(63.9)		
Moderate	7(25.9)	13(36.1)		
Poor	0(0.0)	0(0.0)		
Defecation during pregnancy, n (%)				1.00#
Good	23(85.2)	30 (83.3)		
Moderate	4(14.8)	6(16.7)		
Poor	0(0.0)	0(0.0)		

# P-value of Fishers exact test. * value of Mantel-Haenszel Chi-Square.
