# Supplementary material for: The Relationship between Constitution of Traditional Chinese Medicine in the First Trimester and Pregnancy Symptoms: A Longitudinal Observational Study
Source: Evid Based Complement Alternat Med. 2016 Mar 20;2016:3901485. doi: 10.1155/2016/3901485 (PMC4818819; doi:10.1155/2016/3901485)
Supplement: Supplementary file 1 — Appendix 1: multiple logistic regression analysis (vomiting was considered as independent factor). Appendix 2: stratified analysis by age subgroups (less than 30 years and greater or equal to 30 years). [file 3901485.f1.zip › description.docx]

Chinese medicine has always attached importance to cognition and research on individual differences in human life status. In the Yellow Emperor’s Book of Internal medicine (i.e., Nei-Jing), the earliest medical book in china, described individual differences as body quality nature or body builds, and provided a detailed classification of individual constitutional differences based on the Yin-Yang theory, the five elements (i.e., Wu-Xing), body posture, functional character, and mental characteristics. Zhang Zhongjing, a famous specialist of Chinese medicine in the Eastern Han Dynasty, classified individual differences in human health according to the relationship between constitution and the occurrences, nature, development, and prognosis of diseases. Gradually, more doctors of Chinese medicine featured the term constitution, which was found to be closely related to diseases and to be an important form of the expression of human life status. Constitution was described as an integrated, metastable, and natural specialty in morphosis, physiological functions, and psychological conditions formed on the basis of innate and acquired constitution. This concept combines the internal and external environments of individuals and gives full expression to an outlook known as “harmonization between soma and spirit”. Chinese medicine believes that individuals have diverse constitutions, which serve as the internal basis of human suffering from diseases. In China, constitution has been used in the discovery of underlying disease, as well as in individualized diagnosis and treatment for thousands of years [1]. The identification of constitution also can be used as the basis to provide guidance on dietary and lifestyle for sub-health population, who generally were not found abnormal by modern medical science but could be found to be unbalanced constitution (i.e., disharmony). More recently constitution also has been explored in relation to a specific disease category [2, 3], metabolism [2], and lifestyle behavior [4].

1. J. Wang, T. Wang, Y.S. Li, Y.F. Zheng, L.R. Li, and Q. Wang, "Research on constitution of Chinese medicine and implementation of translational medicine," *Chin J Integr Med, vol.* 21, no. 5, pp. 389-393, 2015.

2. C.H. Lee, T.C. Li, C.I. Tsai, et al., "Association between Albuminuria and Different Body Constitution in Type 2 Diabetes Patients: Taichung Diabetic Body Constitution Study," *Evid Based Complement Alternat Med, vol.* 2015, Article ID 603048, 2015.

3. C.I. Tsai, Y.C. Su, S.Y. Lin, I.T. Lee, C.H. Lee, and T.C. Li, "Reduced health-related quality of life in body constitutions of yin-xu, and yang-xu, stasis in patients with type 2 diabetes: taichung diabetic body constitution study," *Evid Based Complement Alternat Med, vol.* 2014, Article ID 309403, 2014.

4. Y. Zhu, Q. Wang, Z. Dai, et al., "Case-control study on the associations between lifestyle-behavioral risk factors and phlegm-wetness constitution," *J Tradit Chin Med, vol.* 34, no. 3, pp. 286-292, 2014.
